# Changes in mental health levels among Chinese physical education college students from 1995 to 2019

**DOI:** 10.3389/fpsyg.2022.1034221

**Published:** 2022-11-30

**Authors:** Fengzhan Li, Jinxiao Liu, Fanshuo Qiu, Huitao Liu, Sufei Xin, Qun Yang

**Affiliations:** ^1^Department of Military Medical Psychology, Air Force Medical University, Chinese People’s Liberation Army (PLA), Xi’an, China; ^2^Department of Psychology, School of Educational Science, Ludong University, Yantai, China

**Keywords:** physical education college students, SCL-90, mental health, cross-temporal meta-analysis, meta-analysis

## Abstract

To explore the changes in the mental health levels of Chinese physical education college students, the present study conducted a cross-temporal meta-analysis of 43 papers that adopted the Symptom Checklist 90 (SCL-90) from 1995 to 2019. The results showed that the average scores of the seven SCL-90 factors were negatively correlated with the year of data collection. The socioeconomic indicators (GDP, per capita GDP and household consumption level) were significantly negatively correlated with the eight dimension scores of the SCL-90 (except for phobic anxiety). The mean effect sizes of the sex differences in the seven dimension scores (except depression and phobic anxiety) were lower than the small effect size. In conclusion, Chinese physical education college students’ mental health levels have increased in the past 25 years. This phenomenon may be related to Chinese socioeconomic growth, the implementation of national sports policies, and the provision of mental health education for college students. In addition, although the increasing trend in the mental health level of female students was more obvious, there were no significant sex differences.

## Introduction

Studies on the mental health of college students in China began in the 1990s and have been widely performed by a host of researchers in recent years. As a special group of college students, physical education college students have distinct characteristics from general college students (Tian, 2013). On the one hand, similar to general college students, they need to learn theoretical and cultural knowledge. On the other hand, they must complete a series of athletic training assignments during college to further improve their specific sports skills. A study has shown that the particularity of learning and lifestyle may have some effect on the mental health status of physical education college students (Liu, 2009). Additionally, the level of mental health of physical education college students may directly affect their learning quality and career status; that is, a good mental health status guarantees their personal development. Therefore, it is of great significance to study the mental health status of physical education college students.

Many researchers have carried out a series of studies related to the mental health status of physical education college students; however, the results were quite different. Some studies have found that the mental health status of physical education college students is generally better than that of the general population overall (e.g., Xu et al., 2001; Jiang and Pan, 2005), while other studies have found that the mental health status of physical education college students is worse than that of the general population (e.g., Huang et al., 2000; Zhou and Zhou, 2009). The reasons for the inconsistency of the research results are as follows. First, the participants of these studies were diverse, including physical education students from different types of universities (sports colleges and comprehensive colleges) and different regions (east, middle, and west). Second, there are multiple tools for the measurement of the mental health levels of physical education college students, including the Symptom Checklist 90 (SCL-90), the University Personality Inventory (UPI), and the Psychological Health Inventory (PHI) (Zhou and Zhou, 2009; Chang, 2012; Ma et al., 2015). Finally, the most important and easily overlooked reason for the inconsistency of the findings was that the existing studies were cross-sectional studies; that is, they only investigated the mental health levels of physical education students in a certain period but neglected its potential changes over time.

Moreover, it is well-known that China has the second largest economy in the world, and its GDP (Gross Domestic Product) has been rising over the years. Studies have revealed that socioeconomic status has some effect on changes in mental health (Xin et al., 2019; Zhao et al., 2019). Meanwhile, the strength of China’s comprehensive sports programs has gradually improved since the reform and opening-up. With the implementation of the “*Sports Powerful Country*” and “*Healthy China*” strategies, sports science and education, mass sports, and the sports industry continue to develop in China (e.g., Huang, 2018). For example, the curriculum for and specialization of physical education college students have been optimized (Zhang and Li, 2014; Huang et al., 2016; Zhang, 2020), many new jobs have emerged with the vigorous development of mass sports and the sports industry, and society has an increasing demand for sports talent (Chen and Niu, 2014). Therefore, under this background, how do the mental health levels of physical education college students change over time? The present study answered this question by the method of cross-temporal meta-analysis.

Cross-temporal meta-analysis (CTMA) was first used in an empirical study by Twenge (1997). The basic principle of this method is to find coherency among isolated existing studies in chronological order, making these studies a cross-sectional sampling of historical development. With this method, it is possible to analyze the changes in psychological variables related to a large time span from a macroscopic perspective; that is, this method can reveal the changing trends of psychological variables over time. In China, since Xin and Chi (2008) introduced this method, there have been a large number of studies on college students’ mental health (e.g., Xin et al., 2012, 2019; Zhang et al., 2018). These studies revealed that the mental health levels of different types of college students show an obvious upward trend over time. In addition, mental health is frequently assessed by the SCL-90 which is well-developed and has good psychometric properties (e.g., Derogatis et al., 1973; Wang et al., 1999; Xin et al., 2019). Many previous studies published in different years have adopted this scale to measure the mental health levels of Chinese physical education college students (e.g., Liu et al., 2002; Zhang et al., 2015), which made it possible to examine how mental health levels change over time by the CTMA.

In addition, scholars have conducted many empirical studies on the mental health levels of physical education college students, but they have revealed inconsistent conclusions on sex differences. For example, some studies found that females’ mental health levels were lower than those of males (e.g., Liu et al., 2002). Other studies have shown that there is no significant difference in the mental health levels of different sexes (e.g., Chen, 2000). Therefore, the sex differences in the mental health levels of physical education college students need to be examined by a traditional meta-analysis.

In summary, the present study aimed to explore the changes in the mental health levels of Chinese physical education college students during the past 25 years, as well as their relationship with socioeconomic status, and to examine sex differences.

## Methods

### Literature search

The SCL-90 is an instrument to estimate psychological problems with a five-point rating scale, ranging from 1 (not at all) to 5 (extremely). It has a wide coverage of various mental health disorders and contains nine subscales about mental health (i.e., somatic complaints, obsessive-compulsive symptoms, interpersonal sensitivity, depression, anxiety, hostility, phobic anxiety, paranoid ideation, and psychoticism), with a higher score suggesting a lower mental health level (e.g., Derogatis et al., 1973; Xin et al., 2019). The SCL-90 can provide a relatively comprehensive understanding of mental health status. In addition, the Chinese version of the SCL-90 is the most frequently used tool for measuring Chinese physical education college students’ mental health levels (e.g., Liu et al., 2002; Tang et al., 2013). Thus, this study collected data from original research on the SCL-90 scores of Chinese physical education college students to conduct a cross-temporal meta-analysis.

The sources of literature for the current research were three academic literature databases in Chinese: CNKI, Wanfang, and Chongqing VIP. These databases are frequently used in China and include Chinese journals of sciences and social sciences published after 1985, as well as master’s theses and doctoral dissertations. We used search terms such as “SCL-90,” “college students,” “physical education,” “psychological problems,” and “mental health.” Additionally, we searched five academic literature databases in English, including Elsevier, Wiley, ProQuest, PubMed, and Web of Science, using the same keywords. Only articles that provided the average SCL-90 score, sample size, and publication year were collected. In addition, the year of data collection was coded as 2 years before the publication of the previous study, unless a specific year was mentioned in the article (Twenge, 2000; Xin et al., 2019).

### Inclusion rules

**FIGURE 1:**
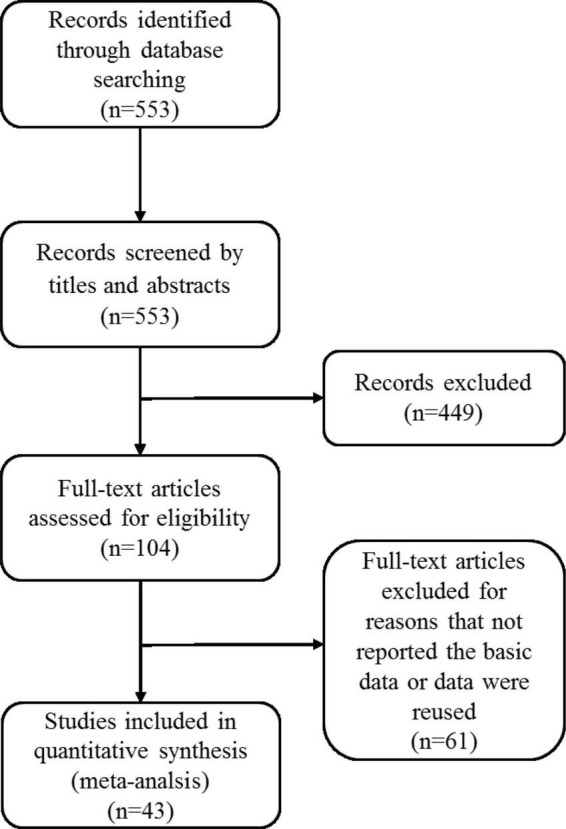
PRISMA flow chart for the screening of studies.

Studies that met the following special inclusion rules were included in the current cross-temporal meta-analysis: (1) studies using the SCL-90 as the tool; (2) studies reporting mean scores and sample sizes for the total or unselected subgroups; (3) studies in which the participants were Chinese physical education college students from routine 4-year facilities; (4) studies in which all participants were from mainland China; (5) studies published before January 2021; and (6) for studies in which the same data were published twice or more, the earliest study was selected. To illustrate the inclusion strategy and the results of the search strategy, a PRISMA diagram is depicted in Figure 1.

As shown in Figure 1 and Table 1, the final sample included 43 studies involving 14,080 physical education college students between 1995 and 2019 (the year of data collection). No English literature met the inclusion criteria. Detailed information for the studies included in the meta-analysis can be found in Table 1.

**TABLE 1:** Description of studies included in the cross-temporal meta-analysis.

First authors	Year of data collection	*N*	Region	Publication class	Somatic complaints	Obsessive compulsive	Interpersonal sensitivity	Depression	Anxiety	Hostility	Phobic anxiety	Paranoid ideation	Psychoticism
Wang	2019	358	3	2	1.25 ± 0.36	1.69 ± 0.53	1.49 ± 0.49	1.49 ± 0.49	1.36 ± 0.43	1.31 ± 0.40	1.25 ± 0.41	1.34 ± 0.42	1.34 ± 0.39
Qian	2017	32	2	2	1.25 ± 0.23	1.32 ± 0.31	1.33 ± 0.24	1.31 ± 0.26	1.32 ± 0.22	1.29 ± 0.26	1.30 ± 0.24	1.29 ± 0.26	1.30 ± 0.26
Liu	2016	145	2	2	1.54 ± 0.56	1.69 ± 0.47	1.58 ± 0.90	1.50 ± 0.59	1.56 ± 0.65	1.51 ± 0.63	1.52 ± 0.57	1.63 ± 0.57	1.55 ± 0.55
Hua	2014	277	3	3	1.31 ± 0.37	1.68 ± 0.53	1.50 ± 0.53	1.42 ± 0.47	1.32 ± 0.49	1.55 ± 0.48	1.30 ± 0.40	1.31 ± 0.35	1.34 ± 0.37
Wang	2014	267	2	2	1.35 ± 0.43	1.60 ± 0.50	1.45 ± 0.38	1.39 ± 0.40	1.30 ± 0.48	1.40 ± 0.23	1.37 ± 0.15	1.24 ± 0.58	1.24 ± 0.53
Fu	2013	70	1	2	1.47 ± 0.38	1.89 ± 0.48	1.69 ± 0.52	1.60 ± 0.49	1.57 ± 0.41	1.68 ± 0.43	1.56 ± 0.60	1.67 ± 0.50	1.56 ± 0.39
Ma	2012	1842	2	2	1.40 ± 0.45	1.72 ± 0.57	1.57 ± 0.55	1.52 ± 0.54	1.45 ± 0.50	1.48 ± 0.55	1.35 ± 0.46	1.47 ± 0.51	1.44 ± 0.48
Liu	2009	146	2	2	1.67 ± 0.58	2.07 ± 0.60	1.83 ± 0.67	1.76 ± 0.61	1.58 ± 0.63	1.67 ± 0.69	1.41 ± 0.52	1.87 ± 0.67	1.71 ± 0.61
Zhang	2009	120	3	2	1.59 ± 0.55	2.02 ± 0.66	1.82 ± 0.60	1.87 ± 0.69	1.64 ± 0.58	1.72 ± 0.67	1.50 ± 0.57	1.68 ± 0.60	1.56 ± 0.50
Fu	2009	50	3	2	1.32 ± 0.48	1.62 ± 0.58	1.80 ± 0.51	1.50 ± 0.59	1.15 ± 0.34	1.48 ± 0.55	1.22 ± 0.32	1.49 ± 0.56	1.23 ± 0.42
Xia	2008	77	2	2	1.45 ± 0.48	1.79 ± 0.55	1.62 ± 0.51	1.60 ± 0.48	1.42 ± 0.41	1.56 ± 0.59	1.30 ± 0.35	1.72 ± 0.57	1.37 ± 0.42
Di	2008	112	2	2	1.28 ± 0.64	1.42 ± 0.85	1.45 ± 0.82	1.47 ± 0.72	1.34 ± 0.69	1.45 ± 0.80	1.31 ± 0.71	1.43 ± 0.76	1.27 ± 0.74
Lv	2007	240	3	3	1.52 ± 0.33	1.75 ± 0.40	1.60 ± 0.36	1.50 ± 0.31	1.49 ± 0.30	1.49 ± 0.30	1.39 ± 0.33	1.48 ± 0.31	1.63 ± 0.36
Zhang	2007	178	1	2	1.44 ± 0.43	1.90 ± 0.51	1.73 ± 0.62	1.57 ± 0.56	1.44 ± 0.46	1.68 ± 0.61	1.38 ± 0.43	1.60 ± 0.54	1.43 ± 0.45
Lei	2007	600	2	3	1.52 ± 0.26	1.56 ± 0.37	1.39 ± 0.28	1.45 ± 0.28	1.69 ± 0.32	1.30 ± 0.26	1.33 ± 0.25	1.71 ± 0.36	1.53 ± 0.28
Wang	2007	245	2	3	1.49 ± 0.46	1.81 ± 0.58	1.63 ± 0.61	1.54 ± 0.58	1.43 ± 0.46	1.53 ± 0.55	1.35 ± 0.41	1.61 ± 0.57	1.42 ± 0.47
Zhang	2006	468	1	2	1.51 ± 0.47	1.89 ± 0.67	1.86 ± 0.69	1.76 ± 0.64	1.59 ± 0.59	1.75 ± 0.75	1.45 ± 0.55	1.72 ± 0.62	1.59 ± 0.55
Huang	2006	538	3	2	1.55 ± 0.53	1.86 ± 0.62	1.80 ± 0.66	1.67 ± 0.63	1.61 ± 0.58	1.72 ± 0.73	1.40 ± 0.51	1.77 ± 0.55	1.59 ± 0.51
Zhao	2006	146	0	3	1.51 ± 0.42	1.86 ± 0.47	1.50 ± 0.46	1.63 ± 0.45	1.58 ± 0.43	1.78 ± 0.55	1.41 ± 0.37	1.67 ± 0.48	1.52 ± 0.41
Xu	2005	673	2	1	1.53 ± 0.49	1.86 ± 0.56	1.72 ± 0.57	1.64 ± 0.56	1.57 ± 0.53	1.68 ± 0.61	1.43 ± 0.50	1.69 ± 0.54	1.58 ± 0.53
Zhang	2005	56	0	2	1.59 ± 0.44	1.79 ± 0.51	1.76 ± 0.49	1.66 ± 0.47	1.41 ± 0.31	1.61 ± 0.37	1.55 ± 0.38	1.66 ± 0.46	1.59 ± 0.48
Fu	2004	1066	1	3	1.35 ± 0.40	1.57 ± 0.47	1.46 ± 0.41	1.42 ± 0.46	1.36 ± 0.42	1.43 ± 0.50	1.31 ± 0.43	1.43 ± 0.47	1.36 ± 0.48
Li	2004	544	2	3	1.36 ± 0.42	1.77 ± 0.63	1.80 ± 0.65	1.65 ± 0.58	1.48 ± 0.44	1.60 ± 0.59	1.35 ± 0.43	1.58 ± 0.56	1.48 ± 0.49
Fu	2004	64	1	2	1.50 ± 0.27	1.93 ± 0.31	1.73 ± 0.40	1.53 ± 0.30	1.45 ± 0.30	1.56 ± 0.41	1.43 ± 0.30	1.65 ± 0.38	1.51 ± 0.38
Sun	2004	256	1	3	1.49 ± 0.46	1.81 ± 0.58	1.63 ± 0.61	1.54 ± 0.58	1.43 ± 0.46	1.53 ± 0.55	1.35 ± 0.41	1.61 ± 0.57	1.42 ± 0.47
Zhao	2004	397	1	2	1.45 ± 0.37	1.79 ± 0.48	1.75 ± 0.50	1.59 ± 0.48	1.52 ± 0.40	1.71 ± 0.58	NA	1.53 ± 0.43	NA
Li	2004	200	2	2	1.60 ± 0.51	1.27 ± 0.36	1.81 ± 0.48	1.49 ± 0.46	1.40 ± 0.40	1.65 ± 0.57	1.21 ± 0.35	1.50 ± 0.61	NA
Chen	2005	1226	2	2	1.72 ± 0.58	2.29 ± 0.61	2.14 ± 0.75	2.12 ± 0.73	1.93 ± 0.67	1.81 ± 0.52	1.45 ± 0.29	1.79 ± 0.63	1.65 ± 0.61
Yang	2004	233	2	2	1.60 ± 0.54	2.05 ± 0.59	2.03 ± 0.63	1.92 ± 0.61	1.68 ± 0.60	1.85 ± 0.72	1.53 ± 0.52	1.86 ± 0.63	1.70 ± 0.56
Shao	2003	58	0	1	1.65 ± 0.44	2.10 ± 0.50	1.99 ± 0.56	1.87 ± 0.58	1.73 ± 0.48	1.82 ± 0.61	1.46 ± 0.61	1.74 ± 0.43	1.70 ± 0.40
Li	2002	151	2	1	1.46 ± 0.46	1.95 ± 0.60	1.82 ± 0.62	1.73 ± 0.61	1.58 ± 0.56	1.72 ± 0.63	1.37 ± 0.42	1.76 ± 0.61	1.59 ± 0.55
Sun	2002	444	0	2	1.56 ± 0.55	2.02 ± 0.63	1.90 ± 0.64	1.77 ± 0.62	1.62 ± 0.57	1.77 ± 0.72	1.42 ± 0.51	1.84 ± 0.63	1.62 ± 0.53
Sheng	2001	852	1	2	1.49 ± 0.49	1.91 ± 0.50	1.98 ± 0.50	1.62 ± 0.47	1.68 ± 0.52	1.90 ± 0.63	1.38 ± 0.54	1.87 ± 0.52	1.43 ± 0.46
Li	2000	108	1	2	1.64 ± 0.52	2.14 ± 0.54	1.99 ± 0.62	2.00 ± 0.65	1.74 ± 0.55	1.92 ± 0.74	1.49 ± 0.48	1.86 ± 0.58	1.58 ± 0.52
Zhao	2000	145	2	1	1.46 ± 0.69	1.51 ± 0.66	1.54 ± 0.71	1.51 ± 0.67	1.52 ± 0.89	1.54 ± 0.72	1.45 ± 0.61	1.50 ± 0.65	1.40 ± 0.60
Liu	2000	223	2	2	1.20 ± 0.44	2.10 ± 0.50	1.99 ± 0.56	1.87 ± 0.58	1.78 ± 0.48	1.82 ± 0.61	1.46 ± 0.41	1.74 ± 0.43	1.70 ± 0.40
Wei	1999	158	2	2	1.41 ± 0.38	1.89 ± 0.59	1.82 ± 0.64	1.69 ± 0.60	1.51 ± 0.46	1.66 ± 0.60	1.34 ± 0.40	1.67 ± 0.51	1.54 ± 0.48
Xu	1999	328	1	1	1.45 ± 0.48	1.85 ± 0.53	1.68 ± 0.52	1.63 ± 0.49	1.41 ± 0.41	1.55 ± 0.55	1.30 ± 0.35	1.74 ± 0.58	1.39 ± 0.40
Sun	1998	152	2	2	1.39 ± 0.37	1.72 ± 0.58	1.69 ± 0.63	1.56 ± 0.53	1.43 ± 0.42	1.57 ± 0.61	1.24 ± 0.35	1.61 ± 0.57	1.44 ± 0.41
Huang	1998	168	1	1	1.53 ± 0.46	2.02 ± 0.64	1.91 ± 0.58	1.81 ± 0.63	1.68 ± 0.58	1.56 ± 0.56	1.52 ± 0.54	1.83 ± 0.67	1.65 ± 0.58
Chen	1998	60	3	1	1.57 ± 0.40	2.17 ± 0.60	2.11 ± 0.69	1.91 ± 0.64	1.69 ± 0.59	1.90 ± 0.59	1.51 ± 0.49	1.96 ± 0.68	1.72 ± 0.26
Dai	1997	94	3	1	1.39 ± 0.29	1.89 ± 0.48	1.89 ± 0.52	1.67 ± 0.39	1.50 ± 0.45	1.89 ± 0.74	1.37 ± 0.36	1.90 ± 0.61	1.71 ± 0.47
Yan	1995	513	4	2	1.38 ± 0.43	1.82 ± 0.54	1.75 ± 0.55	1.59 ± 0.47	1.53 ± 0.51	1.64 ± 0.64	1.36 ± 0.42	1.74 ± 0.51	1.56 ± 0.49

*N* = sample size; region: 0 = no region information, 1 = east, 2 = middle, 3 = west, 4 = mixed; publication class: 1 = core journal, 2 = publication from other academic sources, 3 = dissertations and master’s theses; NA = missing values.

### Coding of control variables

In the present study, the region, publication class and sex ratio in every article were recorded as control variables in the cross-temporal meta-analysis because they may confound with the year. We coded the region into east, middle, west, and mixed (participants in each study were selected from at least two regions). The publication class was divided into three levels (Xin et al., 2019): first class (including the SSCI, SCI, CSSCI, and CSCI), second class (including publications from other sources) and third class (including master’s theses and doctoral dissertations). Publication class was controlled to avoid the effect of publication bias, which refers to the fact that studies with statistically significant results are more likely to be accepted for publication (e.g., Xin et al., 2019). The sex ratio, an important variable for mental health scores, was also recorded in the database and controlled in regression analysis.

### Sources of socioeconomic indicator data

The present study selected the GDP, per capita GDP and household consumption level as indicators to measure China’s socioeconomic environment. These indicators measure a country’s economic situation and development level, with higher values indicating a higher level of economic development. The data of these three indicators came from the China Statistical Yearbook.

### Data analysis strategy

First, for articles that only provided research data without comprehensive study results, the weighted statistics were obtained based on the provided data using the following formula ($\bar{x}$, *x*_*i*_, *n*_*i*_, *S*_*T*_, and *S*_*i*_ represent the combined average, average, study sample size, combined standard deviation, and standard deviation, respectively):


(1) $$\bar{x}=\sum x_{i}\,{\text{n}}_{i}\,/\,\sum {\text{n}}_{i}$$

(2) S=T[∑ni⁢Si2+∑ni⁢(xi-xi¯)2]⁢/⁢∑ni

Second, unlike traditional meta-analysis, the effect size for each study is not computed in CTMA; instead, the key goal of the CTMA is to examine the changes in average scores on psychological measures over time (e.g., Twenge and Campbell, 2001; Xin et al., 2012). Thus, to investigate how Chinese physical education college students’ mental health levels changed over time, correlations were conducted between the mean SCL-90 scores of each study and the data collection year, weighted by the sample size of each study. For the purpose of calculating the magnitude of change in the SCL-90 scores over time, the weighted regression equation and the average standard deviation (*M*_*SD*_) of the individual samples were used in the cross-temporal meta-analysis (Twenge and Im, 2007; Xin et al., 2019). The following weighted regression equation was used to compute the average SCL-90 scores for a particular year: *y* = *Bx* + *C* (where *B* = the unstandardized regression coefficient, *x* = the specific year, *C* = the constant or intercept, and *y* = the predicted mean score). Consistent with previous studies (Twenge and Im, 2007; Xin and Zhang, 2009), we calculated the *M*_*SD*_ by averaging the within-sample *SDs* mentioned in these studies. Notably, this method is more likely to prevent ecological fallacy (Rosenthal et al., 2000; Twenge, 2000).

Finally, to investigate differences in sex, based on traditional meta-analysis, female students were considered the experimental group, and male students were considered the control group. The average effect size *d* was determined by the following formula, where *W*_*i*_ is the study weight, *N*_*i*_ is the total sample size, *d* is the effect size of a single paper, *SD* is the combined *SD* of the experimental and control groups, and *n*_*e*_/*n*_*c*_ and *S*_*e*_/*S*_*c*_ are the sample size and *SD* for the control and experimental groups, respectively.


(3) $$SD=\sqrt{\left[\left(n_{e}-1\right)\,S_{e}^{2}+\left(n_{c}-1\right)\,S_{c}^{2}\right]/\left(n_{e}+n_{c}-2\right)}]$$

(4) d=(M-cM)e/SD

(5) W=i2N/i(8+d)i2

(6) d¯=ΣWdi/iΣWi

## Results

### Changes in the mean scores of the SCL-90 over time

**FIGURE 2:**
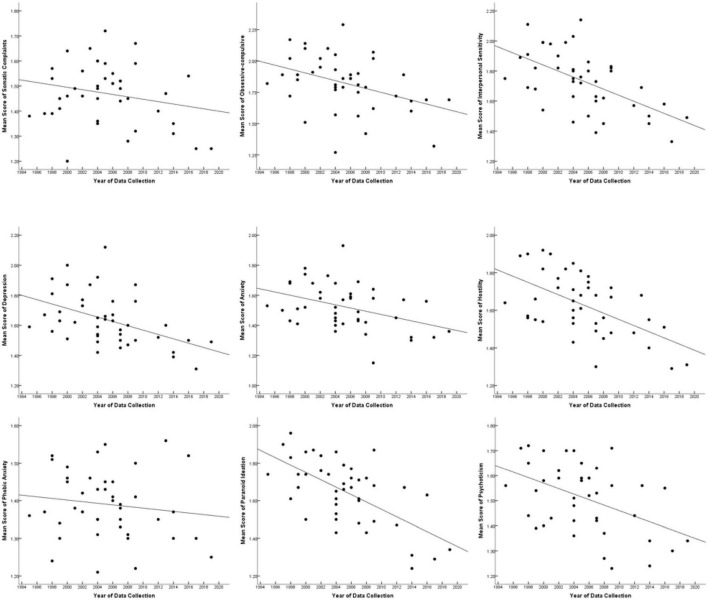
Correlations between the mean SCL-90 scores and year.

Nine scatter plots were created to examine changes in the mental health levels of Chinese physical education college students from 1995 to 2019, with the X-coordinate representing the year of data collection and the Y-coordinate representing the mean score of each dimension (see Figure 2).

The above scatter plots indicated that Chinese physical education college students’ scores on the SCL-90 decreased over time overall, although there were some fluctuations. Moreover, to accurately quantify and describe the changes, we conducted a regression analysis between SCL-90 scores and the year of data collection. As shown in Table 2, the mean scores of seven dimensions were significantly negatively associated with the data-collection year. Similar results were found when controlling for publication class, region and sex ratio and weighting the sample size (see Table 2). In short, it can be seen from the results that Chinese physical education college students’ SCL-90 scores decreased (namely, their mental health levels increased) over the past 25 years.

**TABLE 2:** Relationships between the year of data collection and mental health scores.

Dimension	Without controls		With controls	
	β_1_	95% CI	β_2_	95% CI
Somatic complaints	–0.22	[−0.49, 0.08]	–0.20	[−0.47, 0.11]
Obsessive-compulsive	−0.40**	[−0.62, −0.11]	−0.29^+^	[−0.55, 0.01]
Interpersonal sensitivity	−0.57***	[−0.74, −0.33]	−0.47**	[−0.67, −0.19]
Depression	−0.47**	[−0.67, −0.19]	−0.31*	[−0.56, −0.01]
Anxiety	−0.40**	[−0.62, −0.11]	−0.32*	[−0.56, −0.02]
Hostility	−0.56***	[−0.74, −0.31]	−0.52***	[−0.71, −0.26]
Phobic anxiety	–0.14	[−0.42, 0.18]	–0.19	[−0.46, 0.13]
Paranoid ideation	−0.63***	[−0.78, −0.41]	−0.63***	[−0.78, −0.41]
Psychoticism	−0.46**	[−0.67, −0.17]	−0.37*	[−0.61, −0.07]

^+^*p* < 0.1, *p < 0.05, **p < 0.01, ***p < 0.001; β_1_ is the regression coefficient between SCL-90 scores and year; β_2_ is the regression coefficient between SCL-90 scores and year when the sample size was weighted by controlling for region, publication class, and sex ratio.

### Magnitude of changes in SCL-90 scores

Although the above results showed that Chinese physical education college students’ mental health level increased over time, how much the mental health level increased is not clear. Therefore, the regression equation and the average standard deviation of all the samples were used for analysis, and the effect size (*d*) was calculated to measure the magnitude of change in SCL-90 scores. The regression equation weighted by sample size was used to calculate the average SCL-90 score in the first and last years of the included studies. The results showed that, with the controls, the average scores of nine dimensions decreased by 0.05–0.50 (see “*M*_*change*_” in Table 3) from 1995 to 2019, and the *M*_*SD*_ of the nine dimensions reported for individual samples ranged from 0.43 to 0.57. Thus, the SCL-90 scores decreased by 0.11–0.85 *SD*s in the past 25 years; that is, *d* = −0.11 to −0.85. According to Cohen, 1977 guidelines, the effect size of the dimension of phobic anxiety should be considered lower than a small effect size; the effect sizes of the somatic complaints, anxiety and psychoticism dimensions should be considered between small and medium effect sizes; the effect sizes of the obsessive-compulsive, depression and hostility dimensions should be considered between medium and large effect sizes; and the effect sizes of the interpersonal sensitivity and paranoid ideation dimensions should be considered higher than large effect sizes. Finally, the *d* values (−0.11 to −0.85) of the nine dimensions were transformed to the variance explained by year, and the result showed that the proportion was 0.30–18.09%.

**TABLE 3:** Magnitude of changes in mean SCL-90 scores.

Dimension	*M*_1995_	*M*_2019_	*M*_change_	*M*_SD_	*d*
Somatic complaints	0.91	0.79	−0.12	0.45	−0.27
Obsessive-compulsive	2.40	2.11	−0.29	0.54	−0.53
Interpersonal sensitivity	1.23	0.75	−0.48	0.56	−0.85
Depression	1.36	1.07	−0.29	0.53	−0.54
Anxiety	2.17	1.93	−0.24	0.48	−0.49
Hostility	2.07	1.66	−0.41	0.57	−0.72
Phobic anxiety	2.34	2.29	−0.05	0.43	−0.11
Paranoid ideation	1.52	1.01	−0.50	0.53	−0.94
Psychoticism	2.13	1.94	−0.19	0.47	−0.41

*M*_change_ = *M*_2019_ − *M*_1995_, d = (*M*_2019_ − *M*_1995_)/*M*_SD_, where *M*_SD_ is the average standard deviation.

### Relationship between mental health and socioeconomic indicators

Is there a relationship between increased mental health levels of Chinese physical education college students and socioeconomic changes from 1995 to 2019? The correlation analysis between the mean SCL-90 scores and socioeconomic indicators can answer this question. The results of the correlation analysis are shown in Table 4. After controlling for the sample size, the GDP, per capita GDP and household consumption level variables were significantly negatively correlated with the mean SCL-90 scores, except for phobic anxiety.

**TABLE 4:** The correlations between socioeconomic indicators and mean SCL-90 scores.

Socioeconomic indicators	Somatic complaints	Obsessive-compulsive	Interpersonal sensitivity	Depression	Anxiety	Hostility	Phobic anxiety	Paranoid ideation	Psychoticism
**Year of data collection**									
GDP	−0.37*	−0.41**	−0.58***	−0.49***	−0.41**	−0.59***	−0.17	−0.64***	−0.47**
Per capita GDP	−0.36*	−0.41**	−0.58***	−0.49***	−0.41**	−0.58***	−0.17	−0.63**	−0.47**
Household consumption level	−0.37*	−0.40**	−0.57***	−0.48**	−0.40**	−0.58***	−0.16	−0.63***	−0.46**
**One year before data collection**									
GDP	−0.37*	−0.40**	−0.57***	−0.48***	−0.41***	−0.58***	−0.17	−0.62***	−0.47**
Per capita GDP	−0.37*	−0.40**	−0.57***	−0.48***	−0.42***	−0.58***	−0.17	−0.63***	−0.48**
Household consumption level	−0.37*	−0.41**	−0.57***	−0.48***	−0.41***	−0.58***	−0.17	−0.63***	−0.47**
**Three years before data collection**									
GDP	−0.38*	−0.39**	−0.55***	−0.47**	−0.39**	−0.57***	−0.17	−0.62***	−0.45**
Per capita GDP	−0.38*	−0.39**	−0.55***	−0.47**	−0.39**	−0.56***	−0.16	−0.62***	−0.45**
Household consumption level	−0.36*	−0.39**	−0.54***	−0.47**	−0.39**	−0.57***	−0.18	−0.62***	−0.44**

*p < 0.05, **p < 0.01, ***p < 0.001.

To further illustrate the relationship between socioeconomic changes and the mean SCL-90 scores, lag correlation analysis was carried out; that is, the mean SCL-90 scores were matched and correlated with socioeconomic indicators in two ways: 1 year before and 3 years before data collection. The results found that the correlations between the mean SCL-90 scores and the socioeconomic indicators from 1 year before and 3 years before data collection were basically consistent with the correlations for the year of data collection.

### Sex differences in the SCL-90 scores

To investigate the changes in mental health levels of different sexes, 20 papers containing the mean SCL-90 scores and the *SD*s of males and females were analyzed by cross-temporal meta-analysis. The results found that, with controls, the mean scores of four dimensions on the SCL-90 were significantly negatively associated with the year of data collection among female students. However, male students’ mean scores of all the dimensions were not significantly negatively correlated with the year (see Table 5). That is, female students’ mental health levels had a more obvious increasing trend over time than those of male students.

**TABLE 5:** The changes and the magnitude of changes in different sexes.

Dimension	Male				Female			
	β	*M*_change_	*M*_SD_	*d*	β	*M*_change_	*M*_SD_	*d*
Somatic complaints	–0.07	−0.05	0.42	−0.11	−0.41^+^	−0.16	0.45	−0.35
Obsessive-compulsive	–0.22	−0.25	0.54	−0.46	–0.30	−0.19	0.52	−0.37
Interpersonal sensitivity	–0.37	−0.40	0.55	−0.73	−0.57*	−0.58	0.56	−1.03
Depression	–0.17	−0.21	0.51	−0.41	−0.46^+^	−0.43	0.52	−0.83
Anxiety	–0.05	−0.04	0.47	−0.08	−0.65**	−0.32	0.49	−0.65
Hostility	–0.16	−0.27	0.58	−0.46	−0.53*	−0.30	0.55	−0.55
Phobic anxiety	–0.10	−0.05	0.42	−0.11	−0.48^+^	−0.19	0.46	−0.42
Paranoid ideation	−0.39^+^	−0.34	0.54	−0.64	−0.68**	−0.38	0.51	−0.75
Psychoticism	0.03	0.06	0.47	0.12	–0.26	−0.08	0.47	−0.16

^+^p < 0.1, *p < 0.05, **p < 0.01. β is the regression coefficient between SCL-90 scores and year when the sample size was weighted by controlling for region and publication class.

In addition, we conducted a traditional meta-analysis to analyze the 20 papers that reported mean scores of different sexes (considering male and female students as control and experimental groups, respectively). According to the standards of Cohen (1977), the mean effect sizes of the sex differences in seven dimensions (except the depression and phobic anxiety dimensions) were lower than the small effect size (see Table 6). In other words, there were no significant sex differences in the SCL-90 scores.

**TABLE 6:** The mean effect sizes of sex differences in SCL-90 scores.

Dimension	Somatic complaints	Obsessive-compulsive	Interpersonal sensitivity	Depression	Anxiety	Hostility	Phobic anxiety	Paranoid ideation	Psychoticism
−$\bar{d}$	−0.04	−0.04	−0.18	−0.25	−0.02	0.01	−0.23	0.05	0.14

## Discussion

### Changes in the mental health levels of Chinese physical education college students over time

In the present study, a cross-temporal meta-analysis of 43 papers that adopted the SCL-90 was conducted to examine changes in the mental health levels of Chinese physical education college students from 1995 to 2019. The results showed that scores on seven dimensions of the SCL-90 were significantly negatively correlated with year (except “somatic complaints” and “phobic anxiety”), which indicated that the mental health scores of Chinese physical education college students as measured by the SCL-90 decreased significantly, namely, the overall mental health level of physical education college students in China increased in the past 25 years. The reason that scores of the somatic complaints and phobic anxiety dimensions have not changed significantly over time may be related to the professional characteristics of physical education college students. On the one hand, physical education college students are required to engage in a large number of physical exercise activities, which could help to improve their physical fitness and alleviate somatic complaints. On the other hand, physical education college students also needed to perform many competitive sports activities and daily training to constantly overcome the problem of performance anxiety, which may reduce the development of phobic anxiety symptoms. Moreover, by comparing the data from previous years, the results showed that the somatic complaints and phobic anxiety dimension received lower scores than other dimensions. In terms of the magnitude of change, the overall trend of changes in the mental health levels of physical education college students was consistent with the changing trends of mental health levels among general college students (Xin et al., 2012), poor college students (Zhang et al., 2018) and medical college students (Xin et al., 2019) in China.

### Potential reasons for the changes in the mental health levels of Chinese physical education college students

The present study found that the socioeconomic indicators (GDP, per capita GDP and household consumption level) for 3 years before, 1 year before and the current year of data collection were all significantly negatively correlated with the scores of the eight dimensions of the SCL-90 (except for phobic anxiety). Therefore, it can be concluded that the increase in the mental health levels of Chinese college physical education students may be related to Chinese socioeconomic growth. In fact, some studies have revealed the relationship between socioeconomic status and mental health, which found that a higher socioeconomic level was conducive to mental health status (Maldonadoa et al., 2022) and that a lower socioeconomic level had a negative effect on mental health (Li et al., 2022). Over the past 40 years, China has transformed from having a weak closed economy to an open global economy. China’s economic achievements have attracted worldwide attention, and people’s living standards have improved unprecedentedly. Therefore, people’s sense of happiness and gains are constantly improving, which is bound to promote their mental health status. The results of the present study show that this phenomenon is also reflected in Chinese physical education college students.

In addition, the increase in the mental health levels of Chinese physical education college students may also be related to the implementation of the “*Sports Powerful Country*” and “*Healthy China*” strategies. First, the Chinese government has promulgated many special support policies (i.e., *the Outline for Building a Powerful Nation in Sport*, t*he Guidance on Strengthening Health Promotion and Education*) for sports development (Huang et al., 2018), which has greatly stimulated the vitality of sports development (Li et al., 2020). Under the guidance of special support policies, many government and social funds have poured into the sports industry (Shan et al., 2020). The sports industry is increasingly booming, and the demand for professionals is rising (Wang, 2015), which might alleviate the professional pressure of physical education college students to a certain extent (Chang, 2012; Wang et al., 2018) and then promote the increasing trend in Chinese physical education college students’ mental health levels. Second, with the development and implementation of many policies, such as *the National Standards for Physical Education*, talent training plans for physical education students have been further standardized (e.g., Huang et al., 2016; Yang, 2017). For instance, many colleges and universities have gradually adopted a series of employment-oriented teaching reform measures, which may have strengthened the professional abilities and competitive advantages of physical education college students to a certain extent (Zhang and Li, 2014; Wang et al., 2020) and thereby may have a positive effect on their mental health status.

Another potential reason for the changes in the mental health levels of Chinese physical education college students may be related to the mental health education of college students, which has been continuously strengthened in recent years (Xin and Chi, 2020). Since 2001, the government has successively issued a series of policy documents, such as “*Opinions on Strengthening the Work of College Students’ Mental Health Education in Regular Institutions of Higher Education*” and “*Opinions on Further Strengthening and Improving College Students’ Mental Health Education*,” which have provided necessary guarantees for the sustained and healthy development of college students (Xin et al., 2019). In addition, as a result of receiving consistently mental health education, physical education college students may have a greater awareness of mental health and acquire more skills and strategies in maintaining their mental health levels, such as emotion regulation, self-confidence enhancement, reasonable career planning, etc. Therefore, the provision of mental health education may also help to improve the mental health status of physical education college students to a certain extent.

### Sex differences in the mental health levels of physical education college students

The present study found that the increasing trend of mental health levels in female students was more obvious than that in male students, which might be related to the increasing number of women participating in sports activities in recent years. Many female athletes have obtained excellent achievements in various sporting events, bringing great encouragement to females (Fan et al., 2016). Moreover, the gradually developing sports industry has provided more opportunities for women, which may bring more hope and thereby enhance their mental health status. However, the opportunities of male students have always been relatively abundant, which may be the reason why the changing trend of males’ mental health levels is not as obvious as that of females. Moreover, the results of the traditional meta-analysis found that the difference in the mental health levels between male and female students was not significant, which may be because the living environment, learning environment and problems faced by physical education college students of different sexes are similar.

### Limitations

The present study has the following limitations. First, apart from the SCL-90, there are other tools for testing mental health, e.g., the UPI and PHI (Zhou and Zhou, 2009; Ma et al., 2015). Future research can further explore the underlying mechanisms of physical education college students’ mental health status by comparing the results of cross-temporal meta-analyses that adopted other questionnaires with the current results. Second, the examination of sex differences was based on 20 papers, which may reduce the validity of the results. Future research can confirm the current conclusions until the number of studies is sufficient. Third, although the present study examined the changing trend of physical education college students’ mental health levels in China and provided possible reasons for the trend, it did not further reveal the specific influencing mechanism. Thus, future research should use a longitudinal design and corresponding statistical analysis methods to examine the related influencing factors.

## Conclusion

The present study is the first to explore the changes in mental health levels among Chinese physical education college students by cross-temporal meta-analysis and found that Chinese physical education college students’ mental health levels have increased over the past 25 years. This phenomenon may be related to Chinese socioeconomic growth, the implementation of national sports policies, and the provision of mental health education for college students. In addition, although the increasing trend in the mental health levels of female students was more obvious, there were no significant sex differences.

## Data availability statement

The original contributions presented in this study are included in the article/supplementary material, further inquiries can be directed to the corresponding authors.

## Author contributions

FL: introduction, method, results, and coding. JL, FQ, and HL: method and coding. SX and QY: discussing and revising language. All authors contributed to the article and approved the submitted version.
